# Robotic-assisted total knee arthroplasty improves implant position and early functional recovery for the knee with severe varus/valgus deformity

**DOI:** 10.1186/s12891-024-07203-9

**Published:** 2024-01-24

**Authors:** Yang Yang, Lingjun Jiang, Xiaoxiao Zhou, Xiaobo Zhou, Haixiao Chen, Zhongyi Chen

**Affiliations:** 1 Department of Orthopedics, Taizhou Hospital of Zhejiang Province, Affiliated to Wenzhou Medical University, No. 150 Ximen Street, Linhai City, 317000 Zhejiang Province China; 2 Department of Orthopedics, Shanghai University of Medicine & Health Sciences Affiliated Zhoupu hospital, Shanghai, China

**Keywords:** Robotic-assisted, Total knee arthroplasty, Alignment, Functional recovery, Deformity

## Abstract

**Purpose:**

Robotic-assisted total knee arthroplasty (r-TKA) facilitates precise bone resection and lower limb alignment, yet accuracy and functional recovery for severe varus/valgus deformity is not well-documented. The aim of study was to investigate whether r-TKA improves implant alignment in the coronal and sagittal view and early functional recovery compared to conventional TKA(c-TKA).

**Methods:**

This comparative study included 86 patients with symptomatic knee arthritis who underwent primary TKA at our institution between 1st May and 31th November 2021. Radiological parameters evaluated included hip-knee-ankle angle (HKAA), femoral varus-valgus angle (FVVA), tibial varus-valgus angle (TVVA), posterior tibial slope angle (PTSA), femoral sagittal angle (FSA), posterior condylar offset ratio, and Insall-Salvati index. Operative time, stay length, and complications were reviewed from patient records. The hospital for special surgery (HSS), Visual Analogue Scale (VAS) and knee joint motion range were evaluated at the six-month follow-up.

**Results:**

The c-TKA and r-TKA groups had no significant differences in HKAA (179.73 ± 3.76°, range: 172.10-188.90° vs. 180.53 ± 2.91°, range: 173.30-188.32°, *p* = 0.277), FVVA (96.13 ± 2.61°, range: 90.27-101.52° vs. 96.38 ± 2.23°, range: 90.98-100.95°, *p* = 0.636), and TVVA (88.74 ± 2.03°, range: 83.75–92.74° vs. 89.43 ± 1.83°, range: 85.32–94.15°, *p* = 1.000). Outlier of mechanical alignment incidence (> 3°) was significantly lower in r-TKA compared with c-TKA, 17.50% (7/40) vs. 41.30% (19/46), (*p* = 0.017). PTSA of r-TKA remained significantly lower than c-TKA (*p* = 0.009) in mild-deformity patients. For severe varus/valgus deformity, r-TKA had a significantly lesser HKAA-outlier incidence (*p* = 0.025), PTSA-outlier incidence (*p* = 0.019), and lower PTSA (*p* < 0.001) compared with c-TKA. The r-TKA functional outcome was better than c-TKA regarding HSS (93.12 ± 1.97, range: 90–95, 95%CI:92.11–94.13 vs. 91.33 ± 2.50, range: 85–95, 95%CI:90.20-92.69, *p* = 0.036), and VAS (0.24 ± 0.44, range:0–1 vs. 0.72 ± 0.75, range:0–2, *p* = 0.026), knee joint flexion (118.53° ± 8.06, range: 105–130°, 95%CI:114.39-122.67° vs. 112.22 ± 8.09°, range: 100–130°, 95%CI:108.20-116.24° ,*p* = 0.027) for severe varus/valgus deformity.

**Conclusion:**

r-TKA improved lower-limb coronal alignment, sagittal implant position, and early functional recovery for patients with severe varus/valgus deformity of the knee. r-TKA did not confer substantial advantages over c-TKA in both radiological and clinical outcomes for the mild varus/valgus deformity.

## Introduction

Total knee arthroplasty (TKA) is an optimal treatment for end-stage knee arthritis as it helps alleviate pain, restore knee joint range of motion, and improve quality of life. The demand for primary TKA has risen rapidly over the years. However, the satisfaction rates after conventional TKA(c-TKA) are still < 90% [1, 2] and the dissatisfaction rate following c-TKA is 17–19% [3]. It is of paramount importance to restore proper lower limb alignment and ensure appropriate coronal and sagittal alignment of the implant in TKA for excellent knee function, satisfactory clinical outcomes with high rates of patient satisfaction, and prosthesis longevity [4, 5]. While numerous efforts to improve implant position and mechanical axis alignment in conventional surgical procedures have been made, the malalignment outliner ( > ± 3° from neutral) incidence and soft tissue injury in c-TKA remains high [6], especially for the knee joint with severe varus/valgus deformity.

Robotic-assisted TKA(r-TKA) surgical systems, generally computed tomography (CT) scans-based techniques, have been available for more than 20 years-with the motivation of precise bone cuts, soft tissue safety, precise implant position, and satisfactory lower limb alignment. Moreover, the improvement of r-TKA in lower limb alignment and bone resection has been reported [7–10]. A prospective randomized study compared c-TKA with r-TKA using the ROBODOC surgical system, considering the precision of the joint line and mechanical axis alignment. They found that r-TKA has more precise restoration of the joint line and mechanical axis alignment [11]. Song et al. conducted a prospectively randomized study with 100 patients (50 patients—c-TKA, 50 patients—r-TKA) and reported that r- TKA could provide a precise implant position and reduce the incidence of mechanical axis outliners ( > ± 3° from neutral) [12]. Collins et al. conducted a retrospective study of 72 patients who underwent NAVIO system r-TKA to assess coronal alignment found that 93.3% of patients achieved the coronal alignment goal ( < ± 3° from neutral) [13]. Marchand et al. analyzed the r-TKA to correct the varus deformity or valgus deformity with 7° or greater than 7° and found that only 64%(82/129) of patients with severe varus deformity achieved coronal neutral alignment ( < ± 3° from neutral) and all 7 patients with severe valgus deformity were corrected to neutral alignment. Nevertheless, their study did not illustrate that whether the r-TKA for the severe varus/valgus deformity has a better coronal and sagittal alignment than the c-TKA. The neutral alignment incidence of severe varus/valgus deformity remains unsatisfactory in c-TKA [14]. To the best of our knowledge, the comparison of implant position, in both coronal and sagittal alignment, and early knee joint functional recovery between r-TKA and c-TKA for severe varus/valgus deformity of the knee joint has not been reported. Therefore, this study sought to determine whether r-TKA improves implant alignment and early functional recovery compared to c-TKA.

## Methods

A total of 103 patients with symptomatic knee arthritis who underwent primary TKA at our institution between 1st May and 31th November 2021; The inclusion criteria were as follows: symptomatic knee arthritis, having undergone primary TKA (c-TKA or r-TKA), patient age 50–85 years, and both pre- and post-operation radiographs could be reviewed. The exclusion criteria included: having undergone revision surgery(1 case), missing radiographs(8 cases), underlying knee joint infections(1 case), or patients refusal to be included in the study(3 cases) or lost follow-up(4 cases). Finally, 46 c-TKAs patients and the 40 r-TKAs patients were included in present study.

This study was performed after receiving informed consent from all patients and the approval of the institutional medical ethics committee (K20220305). This study was performed in accordance with the ethical standards as laid down in the 1964 Declaration of Helsinki and its later amendments.

### Surgical procedures

All patients underwent pre-operative CT scans of the lower limb in a supine position, loaded onto the MAKO system for planning. Surgical procedures employed a medial parapatellar approach, with extra-articular placement of femoral and tibial arrays using pins. Osteophytes were removed before articular surface registration. During surgery, bone registration and verification matched the pre-operative CT scan to the computer model, identifying the bony structure. The haptic window allowed for soft tissue preservation. In the sagittal plane, the femoral component flexed 0–5° for optimal sizing and notch prevention, with a posterior slope set at 0–3°. Manual varus and valgus stress tests at 10° and 90° flexion assessed virtual ligament tension in the medial and lateral compartments before bone cuts. Necessary soft tissue releases were performed if balance could not be achieved. All surgeries were performed by the same senior author who is well-experienced in both c-TKA and r-TKA. Neutral mechanical alignment of the lower limb was the objective for both c-TKA and r-TKA. The patella resurfacing procedure was performed if the Outerbridge classification of the patellar cartilage was grade II or worse. The cemented posterior stabilized knee prosthesis was used in both treatment groups. The Scorpio (Stryker, NJ, USA) cemented prosthesis was used for c-TKA, and the Triathlon (Stryker, New Jersey, USA) cemented implant was used for r-TKA. The posterior stabilize implant was used for all patients. A tourniquet was not used in either of the two groups. Mako Robotic-Arm Interactive Orthopedic (RIO) system (Stryker Ltd, Kalamazoo, MI, USA) was used to perform r-TKA.

Postoperatively, both c-TKA and r-TKA patients received the same rehabilitation treatment. Prophylactic antibiotics (cefuroxime 1.5 g bid ivgtt) were administered for 24 h, and rivaroxaban 10 mg qd po was administered for thromboprophylaxis in both groups. On the first postoperative day, all patients performed a weight-bearing walk with the assistance of a walker.

The hospital for special surgery (HSS), Visual Analogue Scale (VAS) and range of motion of the knee joints were evaluated at the six-month follow-up for all patients. The goniometer was used to measure the flexion and extension angle of the knee. Ranawat’s classification was used to assess valgus deformity and Thienpont’s classification was used to evaluate the varus deformity of the knee joint [15, 16]. The minimum clinically important difference (MCID) used for the HSS was 5.41 [17].

### Radiological parameter measurement

The pre-and post-operative radiographs for both c-TKA and r-TKA, including anteroposterior (AP) and lateral views of the knee joint, as well as the standing long leg AP view radiographs of the lower limb were reviewed. The long leg standing AP radiographs were taken using GE digital radiography (General Electric Discovery 650) with full extension of the knee joint and both legs standing, bearing equal weight [18].

**Fig. 1:**
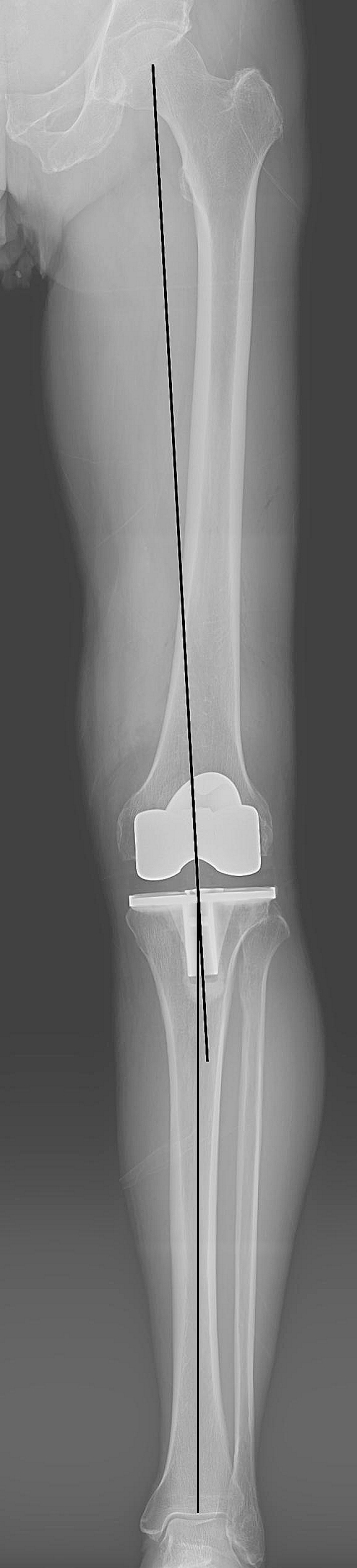
Showing the measurement of the hip-knee-ankle angle which is the angle between the femoral mechanical axis and the tibial mechanical axis

**Fig. 2:**
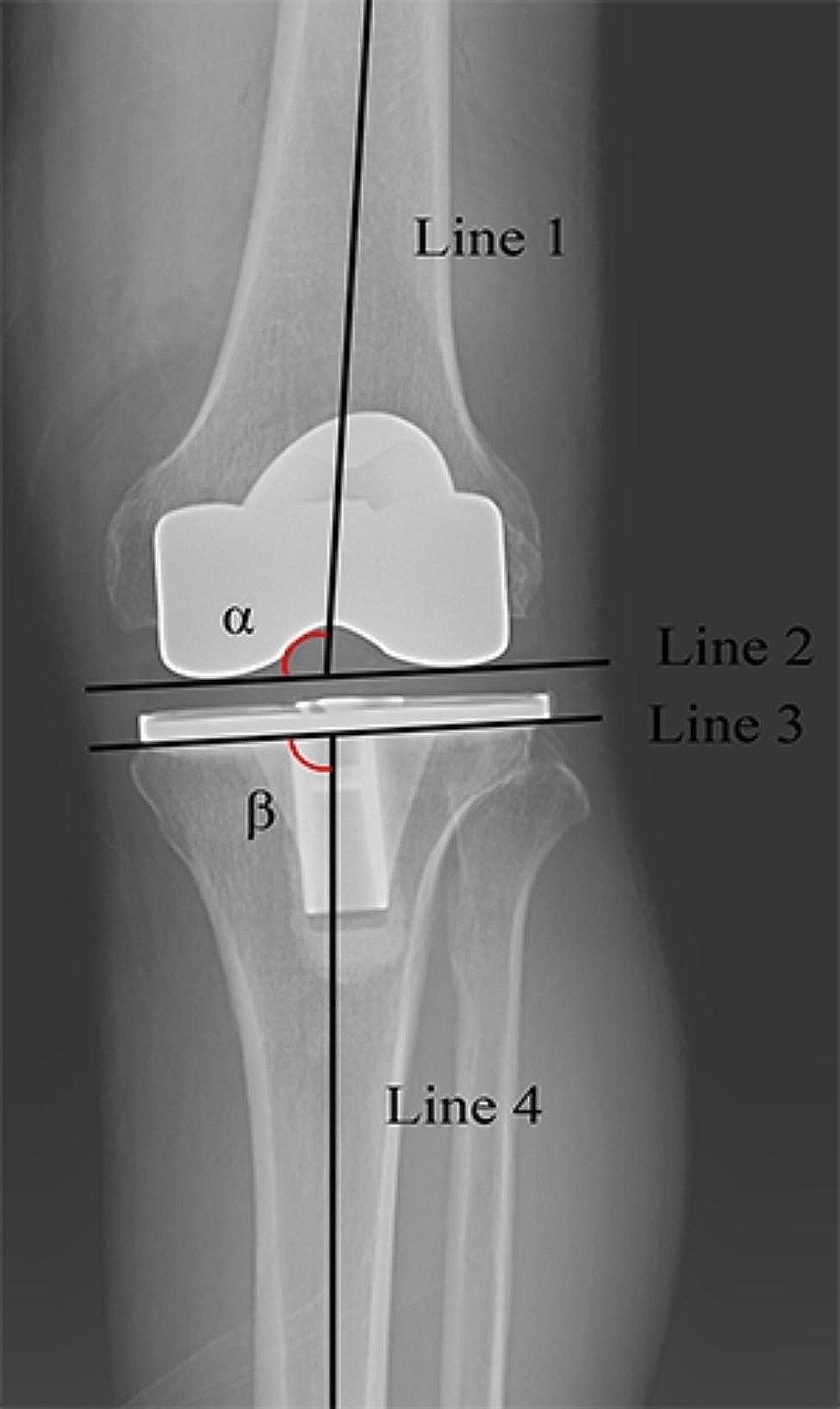
Showing the measurement of the coronal alignment of the prosthesis. Line 1 is the anatomical axis of the femur, line 2 is the tangent line of femoral component, line 3 is parallel to the plateau of tibial component, line 4 is the mechanical axis of tibia. The angle α represents the femoral varus-valgus angle and the angle β is the tibial varus-valgus angle

**Fig. 3:**
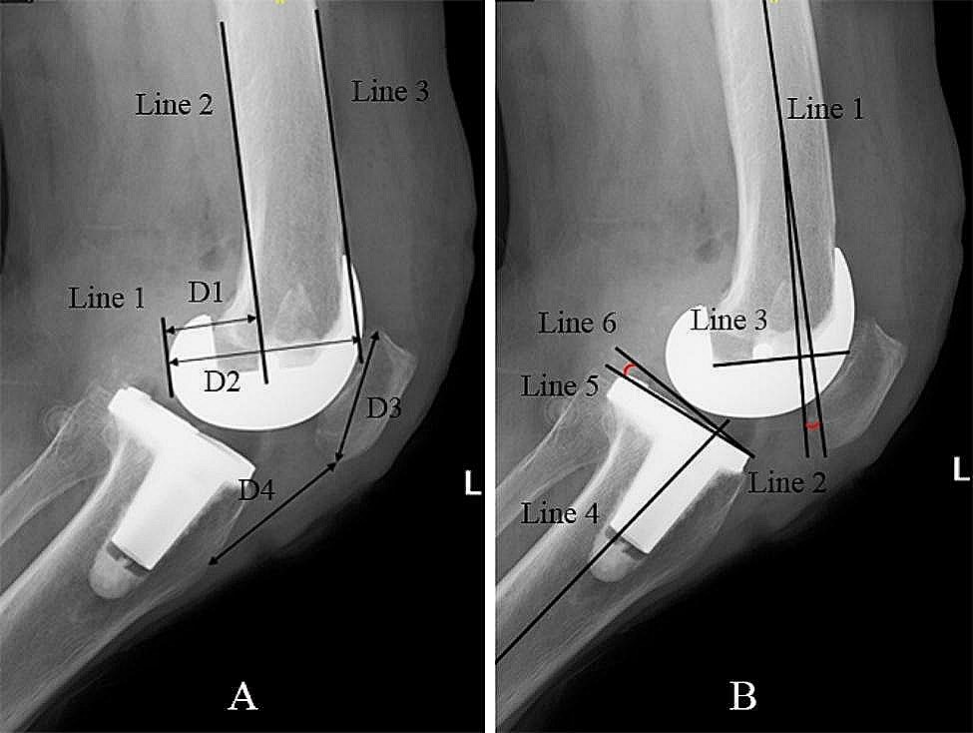
Showing the measurement of sagittal parameters. A, line 3 is the anterior of femur, line 2 is the posterior of femur, line 1 is the tangent line of posterior condylar and parallel to femur shaft. D1 is the distance between line 1 and line 2, D2 is the distance between line 1 and line 3. The posterior condylar offset ratio is calculated as D1 divided by D2. Insall-Salvati index is the value of the length of patellar(D3) divided by the distance between lower poler of the patellar and the tibial tubercle(D4). B, line 1 is the axis of the femur, line 3 is the parallel line of internal surface of the femoral component, line 2 is the perpendicular line of line 3. The angle between line 1 and line 2 is the femoral sagittal angle. Line 4 is the mechanical axis of tibia, line 5 is the tangent line of tibial component surface, line 6 is the perpendicular line of line 4. The angle between line 5 and line 6 is considered as the posterior tibial slope angle

Radiological parameters evaluated in present study included hip-knee-ankle angle (HKAA), femoral varus-valgus angle (FVVA), tibial varus-valgus angle (TVVA), posterior tibial slope angle (PTSA), femoral sagittal angle (FSA), posterior condylar offset ratio (PCOR), and Insall-Salvati index (ISI) [19–21]. The HKAA is the angle between the mechanical axes of the femur and tibia (Fig. 1) [18]. The FVVA is the angle between the anatomical axis of the femur and the tangent line of the distal femoral component surface (Fig. 2). The tibial varus angle is the angle between the mechanical axis of the tibia and parallel line of the tibial component surface (Fig. 2). The PCOR is defined as the greatest distance from the posterior condylar to posterior cortical bone of the femur, divided by the distance to the anterior cortical bone of the femur (Fig. 3A). The ISI is the division ratio of patellar length and distance from the lower pole of the patella to the tibial tubercle (Fig. 3A). The FSA is the angle between the femoral anatomical axis and the perpendicular line of the inside condylar surface of the femoral component (Fig. 3B). The PTSA is the angle between the perpendicular line of the tibial anatomical axis and the parallel line of the tibial component surface (Fig. 3B). The precision of the measurements of the radiological parameters was 0.01 mm and 0.01°. All radiological parameters were measured by two blinded independent senior authors using a Picture Archiving and Communication System.

### Statistical analysis

 The sample size was calculated basing on a published data of the coronal inclination of femoral prosthesis [12]. A minimum of 28 patients were needed in this study to achieve the power of 0.8 with significant value of 0.05. Finally, 46 patients for c-TKA and 40 patients for r-TKA were included. The Kolmogorov–Smirnov test was used to assess whether the data followed a normal distribution. Continuous variables were presented as mean ± SD, and the two-tail student t-test was performed for normally distributed variables. Categorical variables are shown by the number and compared using Chi-square or Fisher’s exact tests when the expected count was < 5. All data were analyzed using SPSS 23.0 (SPSS Inc., Chicago, IL, USA). A *p* < 0.05 was considered statistically significant for all analyses.

## Results

A total of 86 patients who underwent c-TKA (*n* = 46) or r-TKA (*n* = 40) were analyzed. The demographic baseline and general data of all patients are presented in Table 1. No significant differences between the c-TKA and r-TKA groups were found in relation to age, operative sides, sex, BMI, pre-operative varus/valgus of the lower limb, length of stay, and concomitant diseases of hypertension, diabetes, and cardiovascular diseases. There was no significant difference in the main diagnosis (Table 1). Of the 86 patients, 67 exhibited varus deformities, with 6 cases of Thienpont’s type I, 32 cases of type II, 28 cases of type III, and 1 case of type IV. Valgus deformities were observed in 19 patients, including 13 cases of Ranawat’s type I, 5 cases of type II, and 1 case of type III. No significant difference was observed between the two groups in terms of the numbers of severe varus/valgus deformities. The mean operative time of r-TKA was 74.50 ± 22.08 min(range, 45–140), which was similar to the operative time of the c-TKA group (71.74 ± 20.63 min, range: 50-160 min, *p* = 0.55). No psoriatic arthritis, fibromyalgia, systemic lupus erythematosus, lumbar spine disease, or depression was found in present study.

**Table 1:** Demographic data of two groups (mean ± SD, range)

Item	Conventional TKA	Robotic TKA	*P*-value
Patients(n)	46	40	
Left/right(n)	22/24	20/20	0.841
Age(year)	70.28 ± 7.03(53–82)	69.23 ± 7.36(50–83)	0.498
Sex (male/female)	12/34	7/33	0.338
BMI(kg/m^2^)	26.75 ± 3.51(19.51–35.55)	26.47 ± 3.61(21.23–33.92)	0.715
Length of stay(day)	7.76 ± 2.07(5–16)	7.38 ± 2.26(5–16)	0.417
Operative time(min)	71.74 ± 20.63(45–140)	74.50 ± 22.08(50–160)	0.551
Hypertension	33	26	0.502
Diabetes	11	13	0.376
Cardiovascular disease	1	1	1.000
Complications			1.000
Superficial infection	0	1	
Deep infection	0	0	
DVT	2	0	
Main diagnosis			
Osteoarthritis	50	37	
Rheumatoid arthritis	1	2	
Traumatic arthritis	0	1	
Thienpont’s Classification			0.318
Type I and II	23	15	
Type III and IV	14	15	
Ranawat’s Classification			0.350
Type I	5	8	
Type II and III	4	2	

BMI, body mass index; DVT, deep venous thrombosis. Statistically significant *p* < 0.05

There were no significant differences between the c-TKA and r-TKA groups with regard to HKAA (179.73 ± 3.76°, range: 172.10-188.90° vs. 180.53 ± 2.91°, range: 173.30-188.32°, *p* = 0.277), FVVA (96.13 ± 2.61°, range: 90.27-101.52° vs. 96.38 ± 2.23°, range: 90.98-100.95°, *p* = 0.636), and TVVA (88.74 ± 2.03°, range: 83.75–92.74° vs. 89.43 ± 1.83°, range: 85.32–94.15°, *p* = 1.000) (Table 2). No significant difference was found in terms of TVVA (> 3°). While, the incidence of outlier of mechanical alignment (> 3°) in r-TKA group was significantly lower than the c-TKA group(*p* = 0.017), with 17.50%(7/40) mechanical alignment outlier in r-TKA group and 41.30%(19/46) in c-TKA group(Table 2).

**Table 2:** Radiological and functional outcomes of all c- TKA and r-TKA at sixth month follow-up. (mean ± SD, range)

Outcome	c-TKA	r-TKA	*P*-value
HKAA(°)			
Preoperative	174.60 ± 9.15(161.31-204.56)	173.49 ± 9.22(141-192.81)	0.575
Postoperative	179.73 ± 3.76(172.10-188.90)	180.53 ± 2.91(173.30-188.32)	0.277
δ-HKAA	8.40 ± 6.10(0.12–32.46)	9.02 ± 6.34(1.34–35.22)	0.643
HKAA-outlier(± 3°, n)	19/27	7/33	0.017
FVVA (°)	96.13 ± 2.65(90.27-101.52)	96.38 ± 2.23(90.98-100.95)	0.636
TVVA (°)	88.74 ± 2.03(83.75–92.74)	89.43 ± 1.83(85.32–94.15)	1.000
TVVA-outlier(± 3°, n)	8/38	5/35	0.528
PTSA(°)	5.39 ± 3.07(-0.59-12.58)	2.13 ± 3.39(-6.78-9.47)	< 0.001
PTSA-outlier(± 3°, n)	37/9	23/17	0.021
FSA(°)	2.76 ± 3.20(-3.36-11.08)	5.21 ± 4.71(-2.49-16.54)	0.007
PCOR	0.50 ± 0.05(0.38–0.60)	0.50 ± 0.04(0.41–0.61)	0.957
ISI	1.24 ± 0.20(0.7–1.65)	1.27 ± 0.20(0.86–1.77)	0.485
HSS	92.50 ± 2.38(85–96)	93.25 ± 2.12(89–97)	0.129
VAS	0.54 ± 0.72(0–2)	0.25 ± 0.04(0–1)	0.024
Range of motion			
Flexion	113.91 ± 9.71(100–135)	118.63 ± 9.20(105–140)	0.024
Extension	1.52 ± 2.32(0–5)	2.00 ± 2.48(0–5)	0.359

HKAA, Hip-knee-ankle angle; FVVA, Femoral varus-valgus angle; TVVA, Tibial varus-valgus angle; PTSA, posterior tibial slope angle, FSA, femoral sagittal angle; PCOR, posterior condylar offset ration; Insall-Salvati index, ISI. HSS, hospital for special surgery; Visual Analogue Scale, VAS; Statistically significant *p* < 0.05

The sagittal radiological parameters investigated were PTSA, FSA, PCOR, and ISI. The results showed that the r-TKA group had a significant lower PTSA than the c-TKA group (*p* < 0.001). When considering outliers (defined as error > 3° or anterior inclination), the c-TKA group had a higher incidence (37/46) than the c-TKA group (23/40; *p* = 0.021). We also found that the r-TKA group had a significantly higher FSA than the c-TKA group(5.21° ± 4.71°, range: -2.49-16.54, 95%CI: 1.80–3.71 vs. 2.76 ± 3.20°, range: -3.36-11.08, 95%CI: 3.71–6.72; *p* = 0.007). No significant difference in the means of PCOR was found between the two groups (c-TKA: 0.50 ± 0.05, range: 0.38–0.60, 95%CI: 0.48–0.51 vs. r-TKA:0.50 ± 0.04, range: 0.41–0.61, 95%CI: 0.48–0.51, *p* = 0.957). We did not find any significant difference in postoperative patellar height between the c-TKA and r-TKA groups, which had ISI of 1.24 ± 0.20(range, 0.7–1.65; 95%CI:1.18–1.29) and 1.27 ± 0.20(range, 0.86–1.77; 95%CI:1.20–1.33), respectively (*p* = 0.485) (Table 2).

The r-TKA patients got a significant lower VAS at the sixth month follow-up(*p* = 0.024) and a significant larger flexion of the knee joint(*p* = 0.024). While the HSS and extension did not show any difference between two groups(Table 2).

There was no significant difference in the incidence of post-operative complications in this study. Two patients in the c-TKA group developed postoperative muscular vein thromboses which were confirmed by lower extremity vascular ultrasound and rivaroxaban 15 mg bid was administered. One delayed healing of the tibial probe pin insertion occurred in the r-TKA group. The probe pin insertion site was healed after routine dressing change, and cefuroxime axetil tablets 0.25g bid po were administered for 5 days.

Implant alignment and functional outcomes in r-TKA for severe varus/valgus deformity of the knee were analyzed. The results showed that the PTSA of r-TKA was still significantly lower than c-TKA (*p* = 0.009) in mild deformity patients . The c-TKA and r-TKA groups did not differ significantly regarding HKAA, HKAA-outlier (> 3°), FVVA, TVVA, PTSA, PTSA-outlier, FSA, PCOR, ISI, HSS, VAS and range of motion in mild deformity patients. For the severe varus/valgus deformity patients, the r-TKA group had a significantly lesser HKAA-outlier incidence (*p* = 0.025), significantly lower PTSA (*p* < 0.001), and lesser PTSA-outlier incidence (*p* = 0.019) compared to the c-TKA group. The functional outcome of the r-TKA group also has an advantage over the c-TKA with regard to HSS (93.12 ± 1.97, range: 90–95, 95%CI:92.11–94.13 vs. 91.33 ± 2.50, range: 85–95, 95%CI:90.20-92.69, *p* = 0.036), VAS (0.24 ± 0.44, range:0–1 vs. 0.72 ± 0.75, range:0–2, *p* = 0.026), and knee joint flexion (118.53 ± 8.06°, range: 105–130°, 95%CI:114.39-122.67° vs. 112.22 ± 8.09°, range: 100–130°, 95%CI:108.20-116.24° , *p* = 0.027) for severe varus/valgus deformity(Table 3). The difference in HSS between the two groups did not reach the MCID.

**Table 3:** Radiological and functional outcomes of different varus/valgus deformity subgroup at sixth month follow-up.(mean ± SD, range)

		Mild deformity				Severe deformity	
Outcome	c-TKA	r-TKA	*P*-value		c-TKA	r-TKA	*P*-value
HKAA(°)							
Preoperative	175.85 ± 4.44	177.01 ± 5.38	0.405		172.66 ± 13.55	168.72 ± 11.20	0.357
Postoperative	179.95 ± 3.43 (174.80-188.90)	180.87 ± 2.51 (176.12-186.88)	0.287		179.38 ± 4.32 (172.10-187.66)	180.06 ± 3.41 (173.30-188.32)	0.607
δ-HKAA	4.86 ± 2.83 (0.12–11.27)	5.90 ± 3.31 (1.34–11.26)	0.230		13.90 ± 5.77 (5.84–32.46)	13.24 ± 7.07 (2.55–35.22)	0.764
HKAA-outlier(± 3°, n)	8/20	3/20	0.305		11/7	4/13	0.025
FVVA (°)	96.30 ± 2.72 (91.73-101.52)	96.40 ± 2.17 (93.23-100.95)	0.883		95.88 ± 2.59 (90.27–99.36)	96.37 ± 2.37 (90.98-100.85)	0.566
TVVA (°)	88.86 ± 1.75 (83.79–92.03)	89.53 ± 1.85 (85.58–93.18)	0.199		88.54 ± 2.44 (83.75–92.74)	89.31 ± 1.84 (85.32–94.15)	0.266
TVVA-outlier(± 3°, n)	4/24	3/20	1.000		4/14	2/15	0.685
PTSA(°)	4.28 ± 2.37 (0.11–8.86)	2.05 ± 3.45 (-6.78-9.47)	0.009		7.13 ± 3.29 (-0.59-12.58)	2.24 ± 3.40 (-4.55-8.72)	< 0.001
PTSA-outlier(± 3°, n)	19/9	11/12	0.148		18/0	12/5	0.019
FSA(°)	2.30 ± 2.49 (-1.76-8.61)	4.19 ± 4.23 (-2.49-11.16)	0.067		3.47 ± 4.05 (-3.36-11.08)	6.60 ± 5.09 (-0.74-16.54)	0.052
PCOR	0.50 ± 0.05 (0.38–0.60)	0.49 ± 0.04 (0.41–0.56)	0.407		0.49 ± 0.04 (0.44–0.57)	0.51 ± 0.04 (0.45–0.61)	0.259
ISI	1.22 ± 0.22 (0.70–1.55)	1.26 ± 0.17 (0.97–1.61)	0.430		1.26 ± 0.17 (0.97–1.65)	1.27 ± 0.24 (0.86–1.77)	0.915
HSS	93.18 ± 2.07 (88–96)	93.35 ± 2.27 (89–97)	0.782		91.33 ± 2.50 (85–95)	93.12 ± 1.97 (90–95)	0.036
VAS	0.43 ± 0.69 (0–2)	0.26 ± 0.45 (0–1)	0.321		0.72 ± 0.75 (0–2)	0.24 ± 0.44 (0–1)	0.026
Range of motion(°)							
Flexion	115.00 ± 10.63 (100–135)	118.70 ± 10.17 (105–140)	0.213		112.22 ± 8.09 (100–130)	118.53 ± 8.06 (105–130)	0.027
Extension	1.61 ± 2.38(0–5)	2.17 ± 2.53(0–5)	0.415		1.39 ± 2.30(0–5)	1.76 ± 2.46(0–5)	0.644

HKAA, Hip-knee-ankle angle; FVVA, Femoral varus-valgus angle; TVVA, Tibial varus-valgus angle; PTSA, posterior tibial slope angle, FSA, femoral sagittal angle; PCOR, posterior condylar offset ration; Insall-Salvati index, ISI. HSS, hospital for special surgery; Visual Analogue Scale, VAS; Statistically significant *p* < 0.05

## Discussion

**Fig. 4:**
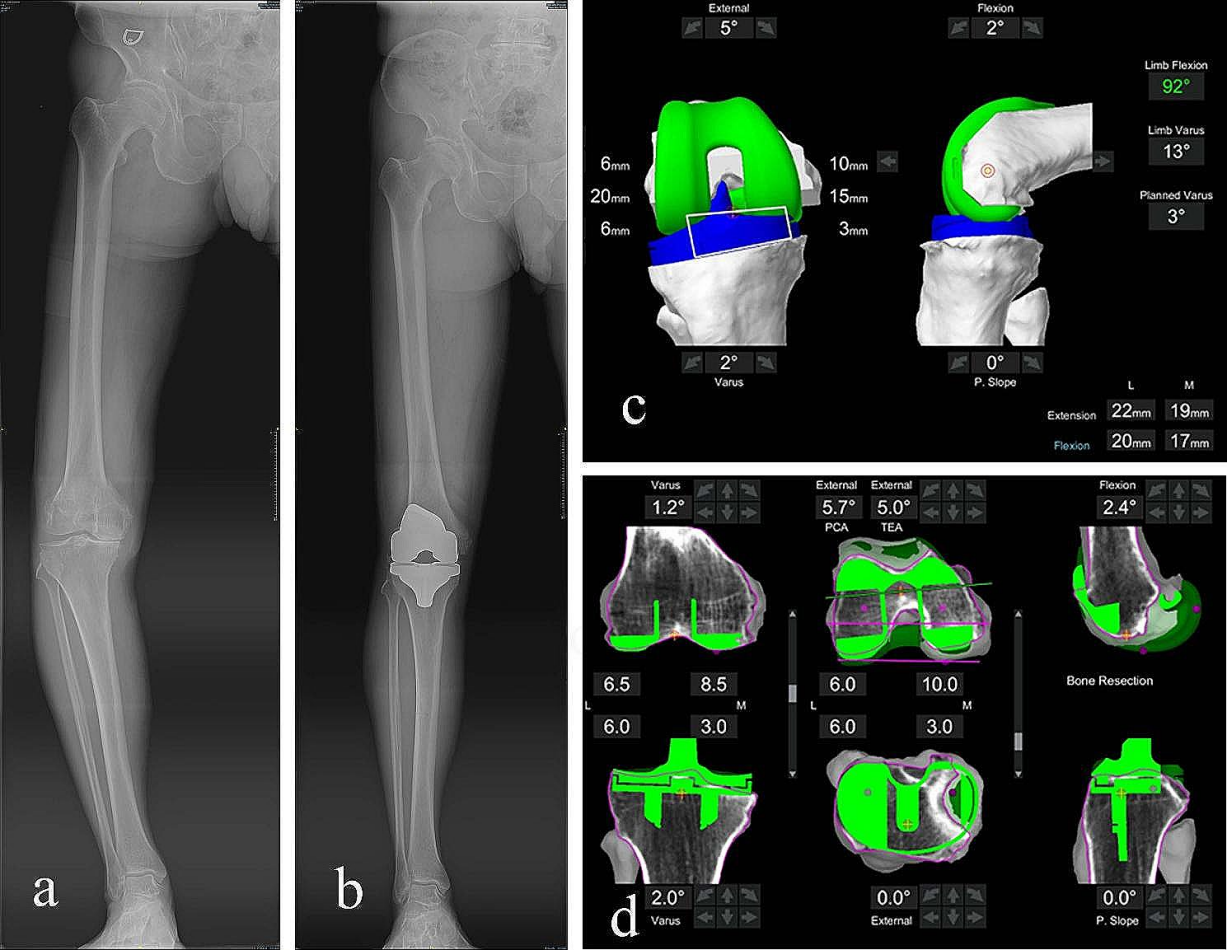
Showing a 66-year-old male patient with a severe varus deformity right knee. The pre-operational full-length AP view of lower limb illustrates a severe varus deformity of the knee joint(**a**); The post-operational full-length AP view of lower limb indicates neutral alignment of coronal alignment(**b**). The Mako Robotic-Arm Interactive Orthopedic (RIO) system was used to balance soft tissues during knee joint flexion (**c**). Intra-operational bone resection planning and performing with the assistance of RIO system(**d**)

The most important finding of present study was that the r-TKA could provide higher precision in both coronal lower limb alignment and sagittal implant position for patients with severe varus/valgus deformity, leading to a better early functional outcome of the knee joint. Restoring the mechanical axis of the lower extremity is one of the primary goals of TKA [22]. Both mechanical alignment and implant position have been shown to determine the implant longevity and clinical outcomes. The existing literature has demonstrated that malalignment of the lower limb mechanical axis > 3° after TKA leads to poor knee function, high incidence of patient dissatisfaction, and short implant longevity [23, 24]. The Mako r-TKA aimed to improve lower limb alignment, achieve precise bone resection and precise implant position, and has been used for several years with satisfactory early clinical outcomes [25]. Several studies have evaluated the accuracy of lower limb alignment and implant position after r-TKA [8, 9, 26]. A cadaver TKA study was previously performed by Hampp et al. to compare the accuracy of bone resection and implant position of r- TKA compared with manual TKA. They found that the robotic-assisted TKA provided a more precise bone resection and implant position [27]. Similarly, another cadaveric study was conducted to assess the accuracy of both coronal and sagittal alignment of the ROSA r-TKA system, and this study concluded that the ROSA r-TKA can improve lower limb mechanical axis alignment and bone resections [28]. Moreover, the ROSA knee system demonstrated precise and consistent outcomes in total knee arthroplasty, employing a collaborative robotic approach to optimize bone resections and ligament balancing without supplanting essential surgical steps [29]. A randomized control trial conducted by Song et al. included 100 patients (50 underwent c-TKA; the rest underwent ROBODOC r-TKA). They also found that there was no significant difference between the two groups in terms of the means of coronal mechanical axis alignment, while no mechanical axis alignment outlier (> 3°) was found in the r-TKA group compared with 24% outliers in the conventional TKA group [12]. Liow et al. conducted a prospective randomized study including 60 patients (31 r TKAs, 29 c-TKAs) to compare the mechanical axis alignment and joint line restoration between r-TKA and c-TKA. They found no mechanical axis alignment outliers (> 3°) in the r-TKA group compared with 19.4% in the conventional TKA group. The joint-line outlier (> 5 mm) of the robotic-assisted TKA group was 3.23%, compared with 20.6% in the conventional TKA group [11]. These studies demonstrated r-TKA is a reproducible technique that is more precise in restoring mechanical axis alignment and produces more precise bone resection than c-TKA. Nevertheless, the accuracy and the early functional outcome of r-TKA for the knee joint with severe varus/valgus deformity has not yet been well documented. In present study, we found that the Mako robotic system can markedly improve the accuracy of both coronal alignment(HKAA-outliers) and sagittal parameters(PTSA and PTSA-outliers), significantly increase the flexion of knee joint and functional outcome(r-TKA vs. c-TKA, HSS: 93.12 ± 1.97 vs. 91.33 ± 2.50, *p* = 0.036) and decrease the VAS at sixth follow-up for severe varus/valgus deformity patients(Fig. 4). The acceptable PTSA was 0–3° for posterior stabilized TKA [22]. The anteroposterior stability of the knee joint would be damaged with an outlier of PTSA. In c-TKA, the sagittal orientation of tibial plateau bone resection was performed using an extramedullary reference, and this was an experience-dependent procedure. This method may result in exceeding the posterior slope of the tibial component. In a series of 29 patients who underwent r-TKA, the mean postoperative tibial posterior slope angle was 1.30° [30], which is similar to our results. In present study, we also found that the r-TKA group had a significantly lager FSA than the c-TKA group. With the real-time assistance of robotic system, the flexion of femoral component could be adjusted to circumvent the air-zone or notch. To avoid femoral notching and improve the equilibrium of both kinematic and biomechanical effects in the sagittal plane, an appropriate sagittal flexion of the femoral component has been proposed [31]. It was interesting that the flexion of the severe deformity knee joint in r-TKA group was found better than the r-TKA group. Every 2° of increased sagittal flexion of the femoral component causes an approximate 1-mm increase in the flexion gap [32]. Hence, an appropriate sagittal flexion of the femoral component may result in a satisfactory flexion of knee joint for severe deformity knee joints.

The r-TKA requires specially prepared preoperative CT scans for planning. A three-dimensional reconstructed image of the knee joint with precise anatomical landmarks facilitates precise bone resection, implant positioning, mechanical alignment, and joint line restoration. Orthopedists can dynamically assess the mechanical alignment, knee stability, extension, and flexion gap during surgery with the assistance of RIO system, enabling real-time adjustment of the bone cut [26]. Intraoperative adjustment of the Mako r-TKA system is a reliable method for performing intraoperative bone cuts with high accuracy [9]. A safety area for bone resection is used for periarticular soft tissue protection [33]. Kayani et al. performed a prospective cohort study consisting of 30 patients who underwent conventional TKA and 30 patients who underwent robotic-assisted TKA, to compare the iatrogenic injury of soft tissue. They found that the r- TKA group had significantly less soft tissue injury compared with the conventional group [34]. Khlpoas et al. performed a cadaveric study to compare the soft tissue injury of robotic-assisted TKA to that of conventional TKA, and found that none of the six robotic-assisted TKAs had ligament disruption, while two of seven cadavers in conventional TKA suffered mild posterior cruciate ligament injury [35]. Adamska et al. conducted a randomized controlled trail which included 215 patients to assess the NAVIO/CORI imageless system for TKA. They concluded that the NAVIO/CORI imageless system can provide an accurate restoration of femoral rotation and a satisfactory clinical outcome [36]. Thus, with the assistance of the robotic system, an over-dissection of the soft tissue could be avoided and more precise lower limb alignment and implant position could be achieved, especially for patients with severe varus/valgus deformity. These major advantages of r-TKA contribute to early functional recovery and decreased acute postoperative pain.

The present study has several limitations to note when considering the findings. First, it was not a randomized controlled trail designed study with a small sample size. Therefore, the selective bias of this study should be considered. Second, we only assessed the lower limb coronal alignment, the coronal and sagittal position of the implant, the patellar height, and PCOR, because these parameters are reproducible on plain radiographs., whereas it is difficult to measure the rotational position of the implant on plain radiographs [37]. A three-dimensional CT reconstruction can provide a more precise implant position measurement, but additional radiations and medical costs need to be considered. Third, we did not exclude patients who were diagnosed with rheumatoid or traumatic arthritis. Finally, the HSS difference of two groups did not reach the MCID in present study, which means the HSS difference between two groups may have no clinically significant, and different implant was used in this study [38, 39]. Therefore, a randomized study with a larger sample size is needed to determine implant longevity and the post-operative functional recovery of knee joints.

## Conclusions

The r-TKA was associated with improved coronal lower-limb alignment, sagittal implant position, and better early functional recovery for patients with severe varus/valgus deformity of the knee joint. While, for patients with mild varus/valgus deformity, r-TKA did not confer substantial advantages over c-TKA in both radiological and clinical outcomes.

## Data Availability

The datasets used and/or analysed during the current study are available from the corresponding author on reasonable request.

## Abbreviations

TKA: Total knee arthroplasty
m-TKA: Mako robotic-assisted TKA
c-TKA: Conventional jig-based TKA
HKAA: Hip-knee-ankle angle
FVVA: Femoral varus-valgus angle
TVVA: Tibial varus-valgus angle
PTSA: Posterior tibial slope angle
FSA: Femoral sagittal angle
PCOR: Posterior condylar offset ratio
ISI: Insall-Salvati index
RIO: Robotic-Arm Interactive Orthopedic
BMI: Body mass index
DVT: Deep venous thrombosis
HSS: Hospital for special surgery
VAS: Visual analogue scale
MCID: Minimum clinically important difference
